# Arecolin

**Published:** 1896-09

**Authors:** 


ARECOLIN. Arecolin is in the market only in the form of the crystalline hydrobromate; in its uncombined state it is a colorless, oily, strongly alkaline, volatile fluid, miscible with water, alcohol, ether, and chloroform in all proportions. It is poisonous, stimulates intestinal peristalsis, and acts as a tænia-fuge.

Prof. Froehner states that arecolin is ten times stronger, as a laxative, than pilocarpin, and fully as powerful as eserin, while its action equals 1000 times that of powdered areca nuts. On the strength of his experience he regards 0.1 gramme (1½ grains) of arecolin, or 100 grammes (3¼ ounces) tincture areca nut, as the maximum dose for horses, and 0.025 gramme (3¾ grains) of the alkaloid, or 250 grammes (say, 8 to 9 ounces) of areca nut, as the maximum dose for oxen. Arecolin is prescribed exclusively in the form of its crystalline hydrobromate.

Arecolin has also been found by Dr. Lavagno to energetically contract the pupil of the eye. In man this effect is produced in three minutes after the introduction into the conjunctival cul-de-sac of one drop of a 1 per cent. aqueous solution of the hydrobromate; it persists for fifteen or twenty minutes, and is entirely dissipated in about one hour. The effect on the ciliary muscle is said to be more rapid, and induces a spasm which lasts from seven to eight minutes. The faculty of accommodation remains normal during the first two or three minutes, then it diminishes somewhat temporarily.

It is reported that the instillation of arecolin hydrobromate never induces cephalalgia or other nervous troubles.

---

The *Veterinary Record* prints the new pension rules for the Veterinary Department of the English army. The rules have received official sanction, and will, it is understood, be shortly published. They are as follows: Veterinary lieutenant-colonel, after thirty years' service, £420 per annum; veterinary lieutenant-colonel, after twenty-five years' service, £400 per annum; veterinary major, after twenty-five years' service, £300 per annum; veterinary major, after twenty years' service, £200 per annum. This scale of pensions is not likely to quicken the flow of promotion, the stagnation in which is paralyzing the zeal and energy of the officers of the department.

